# Systematic Phenotyping and Characterization of the 3xTg-AD Mouse Model of Alzheimer’s Disease

**DOI:** 10.3389/fnins.2021.785276

**Published:** 2022-01-24

**Authors:** Dominic I. Javonillo, Kristine M. Tran, Jimmy Phan, Edna Hingco, Enikö A. Kramár, Celia da Cunha, Stefania Forner, Shimako Kawauchi, Giedre Milinkeviciute, Angela Gomez-Arboledas, Jonathan Neumann, Crystal E. Banh, Michelle Huynh, Dina P. Matheos, Narges Rezaie, Joshua A. Alcantara, Ali Mortazavi, Marcelo A. Wood, Andrea J. Tenner, Grant R. MacGregor, Kim N. Green, Frank M. LaFerla

**Affiliations:** ^1^Institute for Memory Impairments and Neurological Disorders, University of California, Irvine, Irvine, CA, United States; ^2^Department of Neurobiology and Behavior, University of California, Irvine, Irvine, CA, United States; ^3^Transgenic Mouse Facility, University Laboratory Animal Resources, Office of Research, University of California, Irvine, Irvine, CA, United States; ^4^Department of Molecular Biology and Biochemistry, University of California, Irvine, Irvine, CA, United States; ^5^Department of Developmental and Cell Biology, University of California, Irvine, Irvine, CA, United States; ^6^Center for Complex Biological Systems, University of California, Irvine, Irvine, CA, United States; ^7^Department of Pathology and Laboratory Medicine, University of California, Irvine, Irvine, CA, United States

**Keywords:** Alzheimer’s disease, amyloid β-protein, tau, neurofibrillary tangles, amyloid precursor protein, animal model, genetically modified, 3xTg-AD

## Abstract

Animal models of disease are valuable resources for investigating pathogenic mechanisms and potential therapeutic interventions. However, for complex disorders such as Alzheimer’s disease (AD), the generation and availability of innumerous distinct animal models present unique challenges to AD researchers and hinder the success of useful therapies. Here, we conducted an in-depth analysis of the 3xTg-AD mouse model of AD across its lifespan to better inform the field of the various pathologies that appear at specific ages, and comment on drift that has occurred in the development of pathology in this line since its development 20 years ago. This modern characterization of the 3xTg-AD model includes an assessment of impairments in long-term potentiation followed by quantification of amyloid beta (Aβ) plaque burden and neurofibrillary tau tangles, biochemical levels of Aβ and tau protein, and neuropathological markers such as gliosis and accumulation of dystrophic neurites. We also present a novel comparison of the 3xTg-AD model with the 5xFAD model using the same deep-phenotyping characterization pipeline and show plasma NfL is strongly driven by plaque burden. The results from these analyses are freely available via the AD Knowledge Portal (https://modeladexplorer.org/). Our work demonstrates the utility of a characterization pipeline that generates robust and standardized information relevant to investigating and comparing disease etiologies of current and future models of AD.

## Introduction

Alzheimer’s disease (AD) is an age-related neurodegenerative disorder characterized by progressive memory deficits that affects more than 6 million Americans and more than 30 million individuals worldwide (Alzheimer’s Association Report, 2021). Currently, no cure exists and any therapies are palliative in nature with limited benefit to disease progression or pathogenesis. The two hallmark pathologies observed in the AD brain are extracellular plaques composed mainly of the amyloid-beta peptide (Aβ), and intraneuronal neurofibrillary tangles (NFTs), composed primarily of hyperphosphorylated tau protein (DeTure and Dickson, 2019). It is not yet fully understood why these pathologies arise in the aged brain and identifying the underlying and likely multiple mechanism(s) for their development remains a key goal. The accumulation of plaques and NFTs is associated with a cascade of events that ultimately results in extensive synaptic and neuronal loss, which is thought to underlie the memory deficits observed in patients. Neuroimaging and clinical data point to the appearance of cortical plaques as an early predictor of AD, followed by NFTs, brain atrophy, and then clinically detectable impairments (Soria Lopez et al., 2019). Notably, pathology initiates in discrete brain regions and then spreads, with plaques first appearing in the cortex, while NFTs initially accumulate in the entorhinal cortex and hippocampus.

Animal models of AD are valuable resources to investigate mechanisms of AD pathogenesis. Indeed, many observations made using mouse models of AD recapitulate those found in human tissue or imaging experiments, and *vice versa*. However, successful therapies developed and tested in these animal models have been universally unsuccessful in human clinical trials, prompting a reassessment of the development, use and interpretations of data acquired from such models (Veening-Griffioen et al., 2019; Dallemagne and Rochais, 2020). Several issues warrant consideration. First, the vast majority of animal models are based on familial forms of AD, which are rare and inherited in an autosomal dominant fashion, rather than sporadic/late-onset AD which comprises ∼98% of AD cases (Drummond and Wisniewski, 2017). Second, most animal models develop only one of the two hallmark pathologies – either plaques or tangles – while in humans both are required for a transition to AD (Myers and McGonigle, 2019). Third, and perhaps most striking, many existing rodent models that produce abundant AD-related pathologies fail to develop the stark synaptic and neuronal loss observed in clinical AD patients (Drummond and Wisniewski, 2017). However, recent developments in newly generated animal models of AD have recapitulated substantial synaptic and neuron loss, notably the R962-hTau transgenic rat line (Malcolm et al., 2019) and the P301S/E4 transgenic mouse line (Shi et al., 2017), by incorporating FTD-linked tau mutations.

To begin to address these shortcomings as well as meeting the critical need for new animal models of late-onset AD (LOAD), the NIH/NIA established Model Organism Development and Evaluation of Late-onset Alzheimer’s Disease (MODEL-AD^1^). A key goal of MODEL-AD is to develop new strains of genetically modified mice that have combinations of genetic variants associated with increased risk for LOAD in humans. Doing so will allow investigation of the relative contribution of LOAD genetic risk variants to development of AD-like pathology in mice, as well as informing as to the pathways and mechanisms that lead to development of LOAD. While better recapitulating LOAD pathogenesis, such mouse models should ideally improve translatability across preclinical testing pipelines (Oblak et al., 2020). An early objective of MODEL-AD is to establish phenotyping pipelines for robust and reproducible analysis of the newly developed mouse models of LOAD at independent sites. For an initial test of one such pipeline, we are performing an in-depth phenotyping of two popular animal models widely used within the AD research community; 5xFAD mice (Oakley et al., 2006; Forner et al., 2021) and 3xTg-AD mice (Oddo et al., 2003). In addition to validation of the phenotyping pipeline, the results of these analyses will better inform the AD field about the various pathologies that arise, and at which ages and in which brain regions, such that investigators can select the most appropriate model for their hypothesis/experiment. Here, we report the results of our in-depth analysis of the 3xTg-AD model and further validate the use of a robust and standardized phenotyping pipeline to characterize the new animal models combining LOAD-associated genetic variants that are generated by MODEL-AD.

The 3xTg-AD mouse [Tg(APPSwe,tauP301L)1Lfa *Psen1^TM 1Mpm^*/Mmjax] was developed in 2003 and features three familial AD mutations: the Swedish *APP* mutation (KM670/671NL), the *PSEN1* M146V mutation, and the *MAPT* P301L mutation (Oddo et al., 2003). Expression of the multi-copy human *APP* and *MAPT* (TAU) transgenes are regulated by a mouse *Thy1* minigene (Caroni, 1997) while expression of mouse *Psen1* with the M146V mutation is controlled by the cognate mouse *Psen1* locus (Guo et al., 1999). 3xTg-AD mice are usually utilized as homozygous for both the transgene insert and the *Psen1* M146V familial AD mutation. Our initial report of the 3xTg-AD model described development of age-related and progressive amyloid and tau pathologies, with extracellular plaques first appearing at 6-months of age, although replicating the appearance of plaques in 3xTg-AD mice before 12-months of age has been challenging in recent years, followed by NFTs becoming apparent at 12-months of age (Oddo et al., 2003). Additionally, 3xTg-AD mice have also displayed localized neurodegeneration, synaptic impairment, and cognitive deficits at 6 months of age (Drummond and Wisniewski, 2017). The attractive combination of both plaque and tangle development makes the 3xTg-AD mouse regarded as a complete transgenic mouse model of AD pathology (Myers and McGonigle, 2019). Since its original characterization nearly two decades ago, drift has occurred in the phenotypes seen in these mice. This might have occurred due to segregation of alleles in the mixed strain background (combination of C57BL/6, 129/X1 and 129S1), or changes in the transgene copy number within the single site of integration (Goodwin et al., 2019), or other factors. As one of the most widely used animal models in AD studies, we have therefore used the 3xTg-AD mouse as a reference model for the deep-phenotyping pipeline of MODEL-AD to characterize changes in long-term potentiation (LTP), neuropathology, and biochemistry across its lifespan (4, 12 and 18 months of age), and to explore the pathology within the current model compared to our original report. We have also conducted a direct comparison with the 5xFAD model using the same deep-phenotyping pipeline. The results of these systematic phenotyping analyses are freely available via the AD Knowledge Portal^2^ and should be of broad use to the AD scientific AD research community. They also demonstrate the utility of the phenotyping pipeline in providing robust and standardized information relevant to assessing LOAD etiology within current and future models of AD.

## Materials and Methods

### Animals

All experiments involving mice were approved by the UC Irvine Institutional Animal Care and Use Committee and were conducted in compliance with all relevant ethical regulations for animal testing and research. All experiments involving mice comply with the Animal Research: Reporting of *in vivo* Experiments (ARRIVE) guidelines, which are specifically addressed in the Supplementary Material.

### Environmental Conditions

On average, 4–5 animals were group-housed in autoclaved individual ventilated cages (SuperMouse 750, Lab Products, Seaford, DE, United States) containing autoclaved corncob bedding (Envigo 7092BK 1/8″ Teklad, Placentia, CA, United States) and two autoclaved 2″ square cotton nestlets (Ancare, Bellmore, NY, United States) plus a LifeSpan multi-level environmental enrichment platform. Tap water (acidified to pH 2.5–3.0 with HCl then autoclaved) and food (LabDiet Mouse Irr 6F; LabDiet, St. Louis, MO, United States) were provided *ad libitum*. Cages were changed every 2 weeks with a maximum of five adult animals per cage. Room temperature was maintained at 72 ± 2°F, with ambient room humidity (average 40–60% RH, range 10–70%). Light cycle was 14 h light/10 h dark, lights on at 06.30 h and off at 20.30 h.

### Mice

Homozygous 3xTg-AD [B6;129-Tg(APPSwe,tauP301L)1Lfa *Psen1*^*TM*1Mpm^/Mmjax] mice were obtained from a closed colony maintained in the laboratory of F.M.L. In 2018, this stock was genotyped using SNP markers and shown to be homozygous for 129X1/129S1 alleles at ∼35% of the genome, homozygous for C57BL/6 alleles at ∼50% of the genome and heterozygous for alleles of 129X1/129S1 and C57BL/6 at ∼15% of the genome. At that time, qPCR analysis of the APPSwe and TAU P301L cDNA’s in 3xTg-AD mice from the LaFerla colony and those from Jackson Laboratory (Stock # 34830) showed a similar relative copy number for each cDNA. Experimental and control mice for this study were generated as follows. First, sperm from 3xTg-AD homozygous animals from the LaFerla colony was used to fertilize oocytes from B6129SF2/J mice (Jackson Laboratory, Stock # 101045) and zygotes were transferred to pseudopregnant dams. F1 offspring were genotyped to verify heterozygosity for the co-integrated *Thy1-APPSwe* and *Thy1-TAU P301L* transgene array at ∼ 87.9 Mb on chromosome 2, and the I145V/M146V mutations in *Psen1* on chromosome 12. F1 heterozygous mice were intercrossed to generate F2 offspring that were genotyped to identify animals homozygous for both the transgene array and *Psen1* mutations (i.e., experimental 3xTg-AD homozygotes) or *Psen1*^+^*^/^*^+^ and non-transgenic (i.e., wild-type control). For simplicity, throughout the text, 3xTg-AD homozygous animals are referred to as “3xTg-AD” and closely related non-transgenic B6/129 and *Psen*^+/+^ mice are referred to as “wild-type” (WT) controls. Natural breeding or IVF using gametes from F2 3xTg-AD or WT control mice were used to produce F3 and later generations of 3xTg-AD and WT control mice for experimental analysis. All animals were generated by the Transgenic Mouse Facility at UCI.

### Genotyping

To genotype for the presence of the transgene array and to monitor the relative transgene copy number over each generation, we used hydrolysis probes that hybridize to the APP(Swe) mutation (For 5′-TGGGTTCAAACAAAGGTGCAA-3′, Rev 5′-GATGACGATCACTGTCGCTATGAC-3′, Probe 5′-CATTGGACTCATGGTGGGCGGTG-3′) and hTAU (P301L) mutation (For 5′-GCGGGAAGGTGCAGATAAT-3′, Rev 5′-CTCCCAGGACGTGTTTGATATT-3′, Probe 5′-CCAGTCCAAGTGTGGCTCAAAGGA-3′). To normalize Ct values, the APP and TAU signals were normalized to a signal from amplification of the mouse *ApoB* locus (For 5′-CACGTGGGCTCCAGCATT-3′, Rev 5′-TCACCAGTCATTTCTGCCTTTG-3′, Probe 5′-CCAA TGGTCGGGCACTGCTCAA-3′). To genotype the *Psen1* mutant allele, we used hydrolysis probes to discriminate the WT or mutant *Psen1* allele from the same amplicon (For 5′-CACCCCATTCACAGAAGACA-3′, Rev 5′-CAACCCATAGGCAGGTCAAG-3′ *Psen1* WT probe 5′-TGTCATTGTCATTATGACCATCCT-3′, *Psen1* mutant probe 5′-TCATTGTCGTGGTGACCATC-3′).

### Hippocampal Slice Preparation and Long-Term Potentiation Recording

Hippocampal slices were prepared from male and female 3xTg-AD (five females and five males) and WT (five females and five males) mice at 4, 12, and 18 months of age. These timepoints were chosen based on our previous systematic phenotyping characterization of 5xFAD to allow for direct comparisons of phenotypes in 3xTg-AD mice at these same timepoints. Following isoflurane anesthesia, mice were decapitated, and the brain was quickly removed and submerged in ice-cold, oxygenated dissection medium containing (in mM): 124 NaCl, 3 KCl, 1.25 KH_2_PO_4_, 5 MgSO_4_, 0 CaCl_2_, 26 NaHCO_3_, and 10 glucose. Coronal hippocampal slices (340 μm) were prepared using a Leica vibrating tissue slicer (Model: VT1000S) before being transferred to an interface recording containing preheated artificial cerebrospinal fluid (aCSF) of the following composition (in mM): 124 NaCl, 3 KCl, 1.25 KH_2_PO_4_, 1.5 MgSO_4_, 2.5 CaCl_2_, 26 NaHCO_3_, and 10 glucose and maintained at 31 ± 1°C. Slices were continuously perfused with this solution at a rate of 1.75–2 ml/min while the surface of the slices were exposed to warm, humidified 95% O_2/_5% CO_2_. Recordings began following at least 2 h of incubation.

Field excitatory postsynaptic potentials (fEPSPs) were recorded from CA1b stratum radiatum using a single glass pipette filled with 2 M NaCl (2–3 MΩ) in response to orthodromic stimulation (twisted nichrome wire, 65 μm diameter) of Schaffer collateral-commissural projections in CA1 stratum radiatum. Pulses were administered at 0.05 Hz using a current that elicited a 50% maximal response. Paired-pulse facilitation was measured at 40, 100, and 200 s intervals prior to setting baseline. After establishing a 10–20 min stable baseline, LTP was induced by delivering five ‘theta’ bursts, with each burst consisting of four pulses at 100 Hz and the bursts themselves separated by 200 ms (i.e., theta burst stimulation or TBS). The stimulation intensity was not increased during TBS. Data were collected and digitized by NAC 2.0 Neurodata Acquisition System (Theta Burst Corp., Irvine, CA, United States) and stored on a disk.

### Tissue Collection and Histology Preparation

Mice (*n* = 6 per genotype/age/sex) were euthanized at 4, 12, and 18 months via CO_2_ inhalation and transcardially perfused with 1X phosphate buffered saline (PBS; Sigma-Aldrich, St. Louis, MO, United States). These timepoints were selected to enable a standardized comparison of phenotyping data of the present systematic characterization of the 3xTg-AD mouse model and our previous systematic characterization of the 5xFAD mouse model. For all subsequent analyses, brains were removed with hemispheres separated along the midline. Brain halves were either drop-fixed in phosphate buffered 4% paraformaldehyde (Thermo Fisher Scientific, Waltham, MA, United States) at 4°C over a 24-h period for immunohistochemical staining or micro-dissected (cortex, hippocampus, midbrain) and flash frozen for biochemical analysis. Fixed half brains were coronally sliced at 40 μm using a Leica SM2000R freezing microtome. All brain hemispheres were processed and representative slices (between −2.78 mm posterior and −3.38 mm posterior to Bregma according to the Allen Mouse Brain Atlas, Reference Atlas version 1, 2008) containing hippocampal and cortical regions from each mouse were used for histological staining and stored in 4°C in cryoprotectant.

### Immunofluorescence Staining

A representative slice from each mouse was selected and pooled together in wells containing slices from mice of the same experimental group (e.g., identical genotype, age, and sex) for each combination of immunofluorescence stains. Unless specified, all stains were performed at 20°C. For Thioflavin-S (Thio-S) staining, free-floating sections were washed with 1X PBS three times (1 × 10 min, 2 × 5 min) and incubated for 10 min in 0.5% Thio-S (T1892; Sigma-Aldrich, St. Louis, MO, United States) diluted in 50% ethanol. From this point onward, stained sections were kept in the dark. Sections were rinsed with two 5-min washes of 50% ethanol before a final wash in 1X PBS for 10 min. Following the Thio-S stain, sections were treated with a standard indirect immunohistochemical protocol. Briefly, free-floating sections were immersed in normal blocking serum solution (5% normal goat serum with 0.2% TritonX-100 in 1X PBS) for 1 h before an overnight incubation at 4°C in primary antibodies diluted in normal blocking serum solution.

Brain sections were stained with the following diluted primary antibodies against: ionized calcium-binding adapter molecule 1 (IBA1; 1:2000; 019-19741; Wako, Osaka, Japan), Aβ_1–16_(6E10; 1:2000; 8030001; BioLegend, San Diego, CA, United States), glial fibrillary acidic protein (GFAP; 1:1000; AB134436; Abcam, Cambridge, MA, United States), S100β (1:200; AB41548; Abcam, Cambridge, MA, United States), Fox 3 protein (NeuN; 1:1000; AB104225; Abcam, Cambridge, MA, United States), Ctip2 (CTIP2; 1:300; AB18465; Abcam, Cambridge, MA, United States), lysosome-associated membrane protein 1 (LAMP1; 1:200; AB25245; Abcam, Cambridge, MA, United States), human tau (HT7; 1:1000; MN1000; Invitrogen, Waltham, MA, United States), poly-tau (1:1000; Agilent Dako, Santa Clara, CA, United States), phospho-tau Ser202, Thr 205 (AT8; 1:500; MN1020; Invitrogen, Waltham, MA, United States), phospho-tau Thr217 (pTau217; 1:100; 44-744; Invitrogen, Waltham, MA, United States), parvalbumin (PV; 1:500; MAB1572; Millipore Sigma, Darmstadt, Germany). Brain sections were also incubated in *Wisteria floribunda agglutinin* lectin (WFA; 1:1000; B-1355; Vector Labs, Burlingame, CA, United States).

Heat-induced antigen retrieval was necessary prior to staining sections with antibodies against Ctip2, AT8, and pT217. Sections were incubated in pre-heated citric acid buffer (pH 6.0) at 80°C for 30 min and allowed to cool to 20°C for 25 min. Afterward, sections were rinsed in 1X PBS for 10 min and standard indirect immunohistochemical protocol was subsequently performed.

Amylo-Glo staining was used to confirm plaques with an additional marker. Free-floating sections were washed in 70% ethanol for 5 min and rinsed in deionized water for 2 min before being immersed for 10 min in Amylo-Glo RTD Amyloid Plaque Staining Reagent (1:100; TR-200-AG; Biosensis, Thebarton, SA, Australia) suspended in 0.9% saline solution. Afterward, sections were washed in 0.9% saline solution for 5 min and briefly rinsed in deionized water for 15 s before proceeding with standard indirect immunohistochemical protocol.

### Simplified Gallyas’ Method for Silver Staining

A simplified protocol of Gallyas’ method for silver staining was performed to stain NFTs in coronal brain sections of 3xTg-AD and wildtype mice at all timepoints (Kuninaka et al., 2015). A representative slice from each mouse was pooled together in wells containing slices from mice of the same experimental group (e.g., identical genotype, age, and sex) and were rinsed three times in 1X PBS for 5 min each. Following by a final wash of deionized water for 10 min, sections was submerged in 5% periodic acid for 3 min and then rinsed in deionized water for 5 min. The sections were incubated in silver iodide reagent (4% sodium hydroxide, 10% potassium iodide, 0.035% silver nitrate) chilled at 4°C for 1 min and subsequently washed twice in 0.5% acetic acid for 5 min each before rinsing in deionized water. A developer solution was prepared in advanced, combining a solution of 5% sodium carbonate and another solution of 0.19% ammonium nitrate, 0.2% silver nitrate, and 1% tungstosilicic acid, and 0.14% formaldehyde. The sections were incubated in this chilled developing solution for 15–25 min until they developed a pale brown or gray color, after which the development was stopped by immersion in 0.5% acetic acid for 5 min. Sections were mounted and dehydrated before being coverslipped using Cytoseal mounting medium. Microscope slides were allowed to dry before brightfield microscopy.

### Microscopy and Histological Analysis

Immunostained sections were mounted and coverslipped using Fluoromount-G either with or without DAPI (0100-20 or 0100-01, respectively; SouthernBioTech, Birmingham, AL, United States). For whole-brain stitches, automated slide scanning via a Zeiss AxioScan.Z1 was used with a 10×, 0.45 NA objective. Scanned images were corrected for shading, stitched together, and exported using ZEN 2.3 slidescan software for representative images. High resolution confocal images of immunofluorescence images were also captured using a Leica TCS SPE-II confocal microscope with a 10×, 0.3 NA objective lens and LAS-X software. Max projections of 20× Z-stacks of subiculum and visual cortex regions were imaged per section per mouse and used for both Bitplane Imaris quantification and representative images. High resolution images of sections stained using the simplified Gallyas method were imaged using a Zeiss Axioscope 5 with a 20× objective. Images were exported using AxioVision LE64 and used for both FIJI ImageJ quantification and representative images.

#### Imaris Quantitative Analysis

Confocal images of each brain region were quantified automatically using the spots module within the Imaris v9.7 software (Biplane Inc., Zurich, Switzerland) then normalized to the area of the field-of-view (FOV). Amyloid burden was assessed by measuring both the total Thio-S^+^ plaque number normalized to FOV area and their volume via the surfaces module in Imaris software. Similarly, volumetric measurements (i.e., Thio-S^+^ plaque volume, PNN volume, etc.) were also acquired automatically utilizing the surfaces module on confocal images of each brain region. Quantitative comparisons between experimental groups were carried out in sections stained simultaneously.

#### FIJI ImageJ Analysis

Brightfield microscopy images of silver-stained brain sections were first opened in ImageJ software (Schneider et al., 2012) and converted to 16-bit gray-scale. The threshold feature was adjusted and used to distinguish cells from background, which were analyzed using the analyze particles feature. As a result, the ROI manager includes a summary box containing the total number of cells per image.

### Biochemical Analysis

Micro-dissected hippocampal and cortical regions of each mouse were flash-frozen and processed for biochemical analysis. Samples were pulverized using a Bessman Tissue Pulverizer kit. Pulverized hippocampal tissue separated for biochemical analysis was homogenized in 150 μL of Tissue Protein Extraction Reagent (TPER; Life Technologies, Grand Island, NY, United States), while cortical tissue was homogenized in 1000 μL/150 mg of TPER. This composition of TPER includes 25 mM bicine and 150 mM sodium chloride (pH 7.6) to efficiently solubilize proteins within brain tissue following homogenization. Together with protease (Roche, Indianapolis, IN, United States) and phosphatase inhibitors (Sigma-Aldrich, St. Louis, MO, United States), the homogenized samples were centrifuged at 100,000 × *g* for 1 h at 4°C to generate TPER-soluble fractions. For formic acid-fractions, pellets from TPER-soluble fractions were homogenized in 70% formic acid: 75 μL for hippocampal tissue or half of used TPER volume for cortical tissue. Afterward, samples were centrifuged again at 100,000 × *g* for 1 h at 4°C. Protein levels in the insoluble fraction of micro-dissected hippocampal and cortical tissue were normalized to its respective brain region weight, while protein levels in soluble fractions were normalized to the protein concentration determined via Bradford Protein Assay (Bradford, 1976; Green et al., 2011). Formic acid neutralization buffer was used to adjust for pH prior to running ELISA.

#### Electrochemiluminescence-Linked Immunoassay

Quantitative biochemical analyses of human Aβ soluble and insoluble fraction levels were acquired using the V-PLEX Aβ Peptide Panel 1 (6E10) (K15200G-1; Meso Scale Discovery, Rockville, MD, United States). Plates were prepared and read according to the manufacturer’s instructions. Quantitative biochemical analysis of total tau and phosphorylated tau-231 (pTau231) in soluble fractions were also obtained using the Phospho(Thr231)/Total Tau Kit (K15121D-1; Meso Scale Discovery, Rockville, MD, United States). Finally, quantitative biochemical analysis of neurofilament-light chain (NfL) in plasma was performed using the R-Plex Human Neurofilament L Assay (K1517XR-2; Meso Scale Discovery, Rockville, MD, United States).

### Statistics

Every reported *n* represents the number of independent biological replicates. The sample sizes are similar with those found in prior studies conducted by MODEL-AD (Forner et al., 2021) and were not predetermined using statistical methods. Electrophysiology, immunohistochemical, and biochemical data were analyzed using Student’s *t*-test, one-way ANOVA, or two-way ANOVA via Prism v.9 (GraphPad, La Jolla, CA, United States). Bonferroni–Šídák and Tukey’s *post hoc* tests were utilized to examine biologically relevant interactions from the two-way ANOVA. *p ≤ 0.05, ^**^p ≤ 0.01, ^***^p ≤ 0.001, ^****^p ≤ 0.0001. Statistical trends are accepted at ^#^*p* < 0.10. Data are presented as raw means and standard error of the mean (SEM).

### Data Records

The protocols, data, and results are available via the AD Knowledge Portal^3^. The AD Knowledge Portal is a platform for accessing data, analyses, and tools generated by the Accelerating Medicines Partnership (AMP-AD) Target Discovery Program and other National Institute on Aging (NIA)-supported programs to enable open-science practices and accelerate translational learning. The data, analyses and tools are shared early in the research cycle without a publication embargo on secondary use. Data is available for general research use according to the following requirements for data access and data attribution^4^.

Data can be accessed in an interactive matter at UCI Mouse Mind Explorer (see footnote 1). Data is also available for download from Sage Synapse^5^. For access to gene expression data for 3xTG-AD mice described in this manuscript, see doi: 10.7303/syn26524615^6^. For access to gene expression data for 5xFAD mice described in this manuscript, see doi: 10.7303/syn23628482^7^.

## Results

### 3xTg-AD Mice Demonstrate Age-Associated Long-Term Potentiation Impairments

**FIGURE 1:**
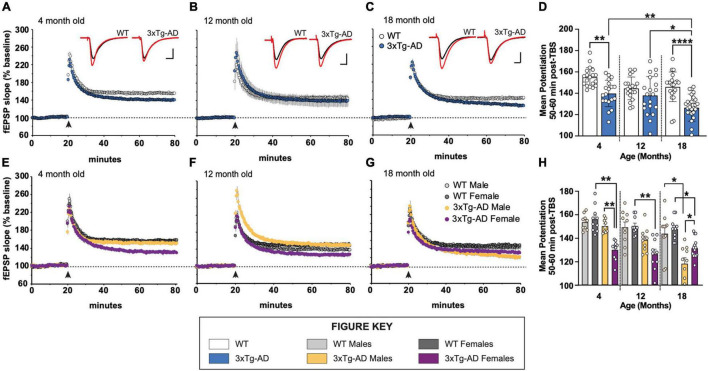
Long-term potentiation (LTP) impairments in 3xTg-AD mice at ages 4, 12, and 18 months in a sex-dependent manner. **(A–C,E–G)** Hippocampal slices were collected from 4-, 12-, and 18-month-old (mo) male and female WT and 3xTg-AD mice and were used to measure LTP in the stratum radiatum in area CA1. Following a 20 min stable recording of field excitatory postsynaptic potentials (fEPSP), LTP was induced by applying five theta bursts (black arrow: each burst containing four 100 Hz pulses with each burst separated by 200 ms) and recording of baseline stimulation was resumed for an additional 60 min. **(D,H)** fEPSP potentiation averaged during the last 10 min of recording in slices from male and female WT and 3xTg-AD mice at ages 4, 12, and 18 mo. *p < 0.05, **p < 0.005, ****p < 0.0001.

We examined long-term potentiation in acute hippocampal slices from 4-, 12-, and 18-month-old male and female 3xTg-AD and WT mice. As early as 4 months of age, there is a marked decrease in LTP 50–60 min post theta burst stimulation (TBS) in stratum radiation of area CA1 in slices from 3xTg-AD mice relative to WT controls (Figures 1A–D). This overall group effect is largely driven by female mice (Figures 1E,H). At 12 months of age, the variability in LTP at 1 h post-TBS is great, however, a deficit in LTP is still present in females, but not males (Figures 1F,H). By 18 months of age, LTP in slices from both male and female 3xTg-AD is-significantly reduced as compared to age-matched control WT slices (Figures 1G,H). Analysis of the mean potentiation during the last 10 min of recording following TBS revealed that there are significant group differences at 4 and 18 months of age (Figure 1D). Moreover, the level of potentiation in slices from 18-month-old 3xTg-AD mice is significantly reduced as compared to slices from 4- and 12-month-old 3xTg-AD.

### Age-Associated Increases in Fibrillar Amyloid Beta Plaque Burden and Size

**FIGURE 2:**
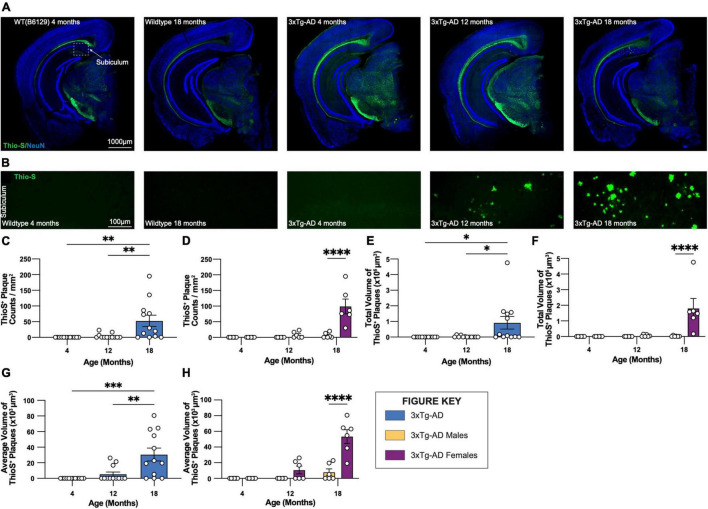
Fibrillar amyloid plaques increase in size and number in 18-month-old female 3xTg-AD mice. 3xTg-AD plaque burden was assessed with Thioflavin-S staining at each time point. **(A)** Representative stitched brain hemispheres of WT and 3xTg-AD mice shown with Thio-S staining at 4- and 18-month, and 4-, 12-, and 18-month timepoints, respectively, counterstained with NeuN. **(B)** Representative confocal images of Thio-S^+^ plaques in subiculum hippocampal regions of WT and 3xTg-AD mice across timepoints displaying increased number of fibrillar amyloid plaques. **(C,D)** Quantification for density of Thio-S^+^ plaques in the subiculum hippocampal region per square millimeter by genotype and sex. **(E,F)** Quantification of total volume of Thio-S^+^ plaques by genotype and sex demonstrating age- and genotype-associated increases in total volume of plaques. **(G,H)** Quantification of average volume of Thio-S^+^ plaques showing an increase in the average volume of a plaque related to age and genotype. *n* = 6 mice per genotype/age/sex. Data are represented as mean ± SEM. *p ≤ 0.05, ^**^p ≤ 0.01, ^***^p ≤ 0.001, ^****^p ≤ 0.0001.

Coronal brain sections from 3xTg-AD and control mice of both sexes at 4-, 12-, and 18-month timepoints were stained with Thio-S for the characterization of fibrillar Aβ plaques (Figures 2A,B). As expected, no plaques are detected in WT control mice. Thio-S plaques are only seen in a subset of female 3xTg-AD hippocampi at 12 months of age and most females by 18 months (Figures 2C,D). Male mice have far fewer plaques, with only a small number of males showing Thio-S^+^ plaques at 18 months. Notably, plaques are not detected outside of the subiculum in any animal (Figure 2A). Consistent with these observations, the total volume of Thio-S^+^ plaques is significantly increased in 18-month-old 3xTg-AD mice compared to other timepoints, an effect largely driven by female 3xTg-AD mice (Figures 2E,F). A similar sex-specific effect is seen with the average volume per Thio-S^+^ plaque (Figures 2G,H), consistent with an increase in plaque size between 12-month and 18-month timepoints.

**FIGURE 3:**
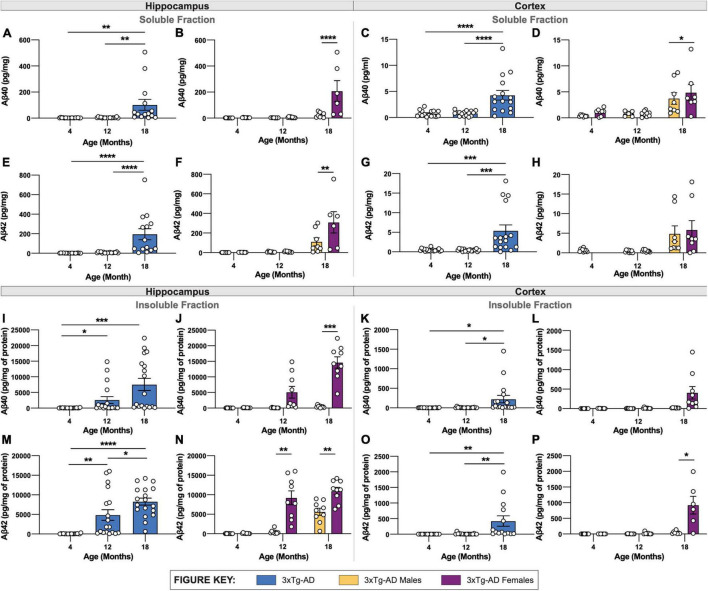
Quantification of Aβ isoforms in 3xTg-AD mice of different age and sex. Aβ was quantified in micro-dissected hippocampi and cortices via Mesoscale Multiplex technology. **(A–H)** Aβ40 and Aβ42 were measured in the soluble fraction of hippocampus **(A,B,E,F)** and cortex **(C,D,G,H)**, respectively, with age-related increases in Aβ40 and Aβ42 shown in hippocampus and cortex of 3xTg-AD mice between sexes. **(I–P)** An age-related increase in insoluble Aβ40 and Aβ42 was also observed in hippocampus **(I,J,M,N)** and cortex **(K,L,O,P)** of 3xTg-AD mice between sexes. *n* = 5–6 mice per genotype/age/sex. Data are represented as mean ± SEM. *p ≤ 0.05, **p ≤ 0.01, ***p ≤ 0.001, ****p ≤ 0.0001.

Aβ40 and Aβ42 were quantified in detergent soluble and insoluble fractions of micro-dissected hippocampus and cortical tissue. The soluble and insoluble forms correspond to recently produced Aβ, and Aβ contained within plaques, respectively. As anticipated, a substantial increase in both Aβ40 and Aβ42 is found in soluble fractions of both hippocampus and cortex in 18-month 3xTg-AD mice (Figures 3A–H). Consistent with plaque load, 18-month-old female 3xTg-AD mice display elevated soluble Aβ40 and Aβ42 in hippocampus compared to 3xTg-AD male mice (Figures 3B,F). Soluble Aβ40, but not Aβ42, is slightly, but significantly increased in the cortices of female 3xTg-AD mice compared to male 3xTg-AD mice (Figures 3D,H).

In tandem with increased plaque density, insoluble Aβ40 and Aβ42 are also increased in hippocampal and cortical regions in 3xTg-AD mice with age (Figures 3I–P). Insoluble Aβ40 and Aβ42 progressively increase between 4-, 12- and 18-month-old 3xTg-AD mice (Figures 3I,M). As with soluble Aβ, there is a clear sex difference in the quantity of insoluble Aβ, with 18-month-old female 3xTg-AD mice showing significantly more Aβ40 and Aβ42 in hippocampal tissue (Figures 3J,N).

### Accumulation of Human Tau and Poly-Tau in Hippocampal Regions

**FIGURE 4:**
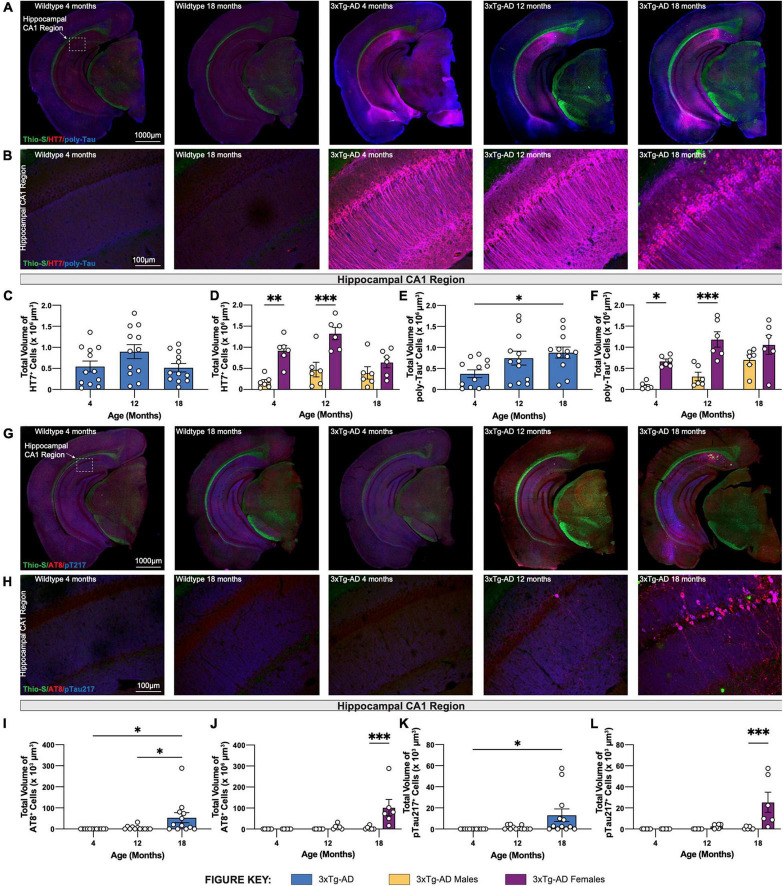
Phosphorylated tau increases in volume in 18-month-old female 3xTg-AD mice. Accumulation of human tau was assessed by immunostaining using HT7 and poly-tau antibody, while phosphorylated tau was assessed using AT8 and phospho-Tau Thr 217 antibody, respectively. **(A)** Representative stitched brain hemispheres of WT and 3xTg-AD mice stained with Thio-S/HT7/poly-tau at 4- and 18-month and 4-, 12-, and 18-month timepoints, respectively. **(B)** Representative confocal images of HT7^+^ and poly-tau^+^ cells in the hippocampal CA1 regions of WT and 3xTg-AD mice across respective timepoints. **(C,D)** HT7 immunostaining reveals age-related changes in total volume of HT7^+^ cells in 3xTgAD mice between sexes. **(E,F)** Poly-tau immunostaining reveals age-related changes in total volume of poly-tau^+^ cells in 3xTg-AD mice between sexes. **(G)** Representative stitched brain hemispheres of WT and 3xTg-AD mice stained with Thio-S/AT8/pT217 at 4- and 18-month and 4-, 12-, and 18-month timepoints, respectively. **(H)** Representative confocal images of AT8^+^ and Thr217^+^ cells in the hippocampal CA1 regions of WT and 3xTg-AD mice across respective timepoints. **(I,J)** AT8 immunostaining reveals age-related changes in total volume of AT8^+^ cells in 3xTg-AD mice between sexes. **(K,L)** Thr217 immunostaining reveals age-related changes in total volume of Thr217^+^ cells in 3xTg-AD mice between sexes. *n* = 5–6 mice per genotype/age/sex. Data are represented as mean ± SEM. *p ≤ 0.05, **p ≤ 0.01, ^***^p ≤ 0.001.

Brain sections from 3xTg-AD mice at 4-, 12-, and 18-month timepoints were stained for human tau using HT7 antibody and combined human & mouse tau protein using poly-tau antibody. As expected, in wildtype mice expression of human tau or somatodendritic accumulation of murine tau is not observed (Figures 4A,B). In contrast, 3xTg-AD mice, that overexpress mutant human tau (P301L), display substantial staining using HT7^+^ and poly-tau^+^ antibodies in the somatodendritic compartment of hippocampal CA1 region neurons (Figures 4A,B). Quantification of the total volume of HT7^+^ cells demonstrates age-related differences in 3xTg-AD mice, increased at 12-months but decreased at 18-months (Figure 4C). This effect is mostly contributed by female 3xTg-AD mice, which have greater total volumes of HT7^+^ neurons compared to male 3xTg-AD mice at 4- and 12-month timepoints. Interestingly, this sex difference diminishes at 18-months of age (Figure 4D). Unlike total volumes of HT7^+^ cells, the total volume of cells staining for poly-tau antibody display an age-associated increase in hippocampal CA1 regions of 18-month 3xTg-AD mice (Figure 4E). Once again, sex differences are evident at 4- and 12-month timepoints as female 3xTg-AD mice exhibit greater total volumes compared to males, an effect that diminishes at the 18-month timepoint (Figure 4F).

**FIGURE 5:**
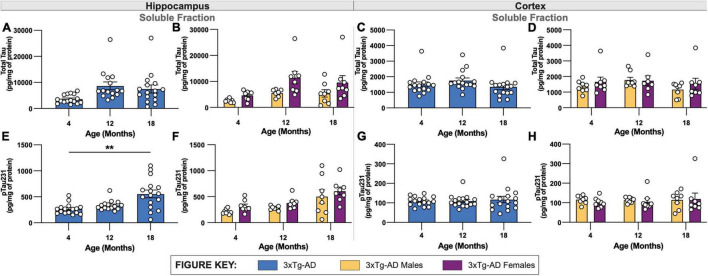
Quantification of total tau and phosphorylated tau in 3xTg-AD mice of different ages and sexes. Levels of total tau and phosphorylated tau were quantified in micro-dissected hippocampi and cortices using Mesoscale Multiplex technology. **(A–D)** Total tau was measured in the soluble fraction of hippocampus **(A,B)** and cortex **(C,D)**, respectively, with no significant differences found between age or sex in either brain regions of 3xTg-AD. **(E–H)** Levels of phosphorylated tau 231 (pTau231) significantly increased with age specifically in soluble fractions of hippocampus **(E,F)** but not cortex **(G,H)** of 3xTg-AD mice. Differences in pTau231 levels were not seen between sexes. *n* = 5–6 mice per genotype/age/sex. Data are represented as mean ± SEM. **p ≤ 0.01.

Similar to the biochemical analysis of Aβ40 and Aβ42, levels of total tau were quantified in soluble fractions of micro-dissected hippocampal and cortical tissue. Mirroring changes in the volume of HT7^+^ immuno-stained cells in hippocampus, minimal age-related increases in total tau levels were found in both soluble hippocampal and cortical tissue of 3xTg-AD mice between 4- and 12-months of age (Figures 5A,C). At 18-months of age, 3xTg-AD mice demonstrated a slight reduction in total tau levels in soluble fractions of both hippocampal and cortical tissue. No sex-associated differences were identified in either hippocampal or cortical tissue of 3xTg-AD brains across all timepoints (Figures 5B,D).

### Age-Associated Accumulation of Phosphorylated Tau Species in Hippocampal Regions

To characterize hyperphosphorylated tau in 3xTg-AD mice, brain sections from each 3xTg-AD mouse at 4-, 12-, and 18-month timepoints were stained for phosphorylated tau species using antibodies against paired helical filament tau phosphorylated at serine 202 and threonine 205 (via antibody AT8). Phospho-tau Thr217 (pTau217) was recently found to be present in the blood plasma of AD patients (Barthelemy et al., 2020; Palmqvist et al., 2020). Therefore, an antibody against pTau217 was also used to detect this post-translational modification in the brain sections of 3xTg-AD mice at each timepoint. No AT8^+^ and pTau217^+^ cells are observed in the brains of age-matched wildtype mice. In contrast, a strong signal to phosphorylated tau was present in the brains of 18-month-old 3xTg-AD mice (Figures 4G,H). Quantification of the total volume of AT8^+^ and pTau217^+^ cells show significant age-related accumulation of phosphorylated tau species in the hippocampal CA1 region of 3xTg-AD mice (Figures 4I,K). Again, accumulation of phospho-tau species is driven largely by female 3xTg-AD mice (Figures 4J,L).

To mirror histological analysis of phosphorylated tau, levels of phosphorylated Tau 231 (pTau231) were also measured in soluble fractions of micro-dissected hippocampus and cortical tissue revealing an age-associated increase hippocampal tissue, but not cortical tissue (Figures 5E,G). No sex-related differences of levels of pTau231 were identified in either brain regions of 3xTg-AD (Figures 5F,H).

### Development of Neurofibrillary Tangles in the CA1 of Aged Female 3xTg-AD Mice

**FIGURE 6:**
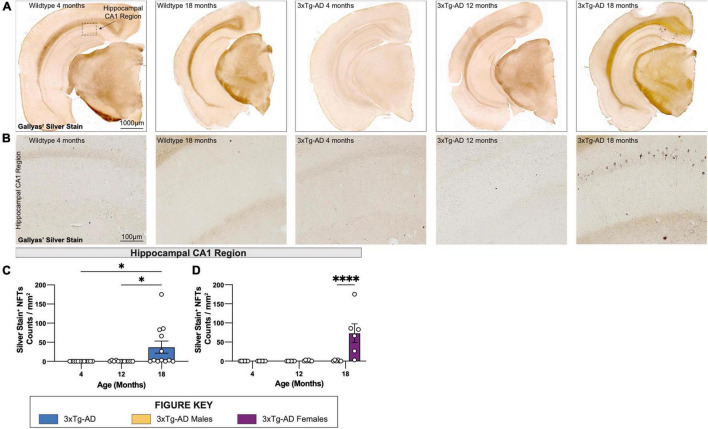
Neurofibrillary tangles increase in number in 18-month-old female 3xTg-AD mice. Accumulation of neurofibrillary tangles (NFTs) was assessed using Gallyas’ silver staining method at each timepoint. **(A)** Representative stitched brain hemispheres of WT and 3xTg-AD mice at 4- and 18-month, and 4-, 12-, and 18-month timepoints, respectively. **(B)** Representative brightfield images of silver stained NFTs in hippocampal CA1 regions of WT and 3xTg-AD mice across each timepoint showing progressive accumulation of NFTs. **(C,D)** Quantification for density of silver stained NFTs in hippocampal CA1 regions per square millimeter by genotype and sex. *n* = 6 mice per genotype/age/sex. Data are represented as mean ± SEM. *p ≤ 0.05, ^****^p ≤ 0.0001.

To visualize NFTs, brain sections from each 3xTg-AD mouse at 4-, 12-, and 18-month timepoints were stained using a simplified Gallyas’ silver stain. As expected, no NFTs are detected in age-matched wildtype controls but are present in female 3xTg-AD hippocampi at 18 months of age (Figures 6A–D), but not in male mice, or in other brain regions (Figures 6A–D).

### Age-Related Gliosis in 3xTg-AD Mice

**FIGURE 7:**
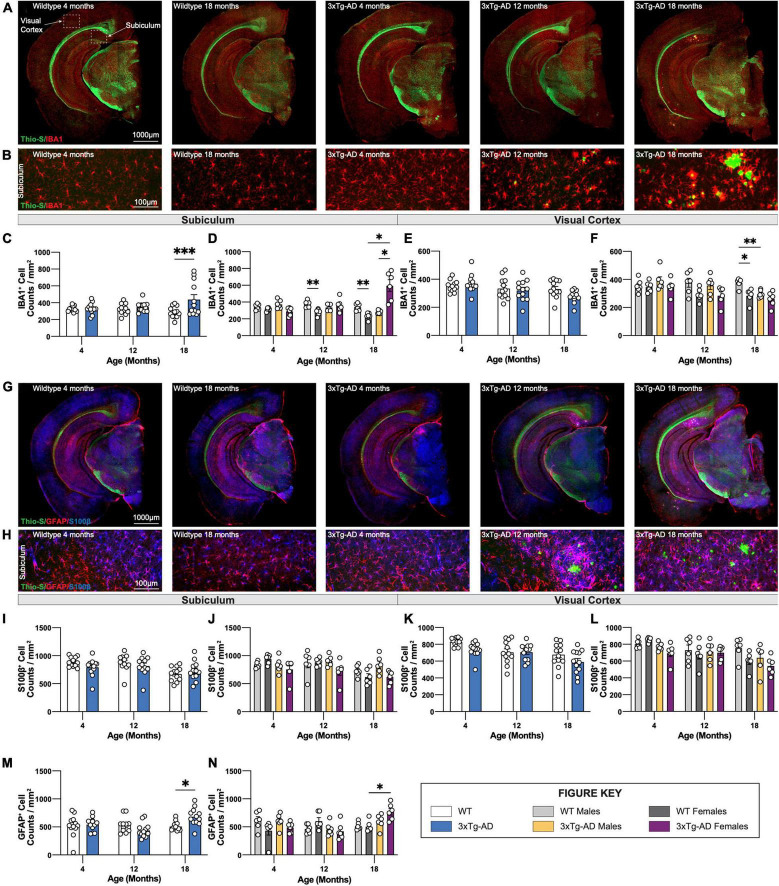
Immunostaining of microglia and astrocytes. Brains of mice at each timepoint were coronally sectioned and immunostained for IBA1, GFAP, and S100β to identify changes in microglia or astrocytes. **(A)** Representative stitched images of brain hemispheres of WT (4-, 18-month) and 3xTg-AD mice (4-, 12-, 18-month) stained with Thio-S/IBA1. **(B)** Representative images of IBA1^+^ cells surrounding Thio-S^+^ in subiculum hippocampal regions of WT and 3xTg-AD mice at indicated timepoints. **(C–F)** Age-related changes microglial density in both WT and 3xTg-AD, and differences between genotypes in subiculum hippocampal and cortical regions. **(G)** Representative images of brain hemispheres of WT (4-, 18-month) and 3xTg-AD mice (4-, 12-, 18-month) stained with Thio-S/GFAP/S100β. **(H)** Representative images of GFAP^+^ and S100β^+^ cells surrounding Thio-S^+^ plaques in subiculum hippocampal regions of WT and 3xTg-AD mice at indicated timepoints. **(I–N)** Astrocyte density as assessed via S100β **(I–L)** and GFAP **(M,N)** staining in the subiculum hippocampal and cortical regions. Age-related changes in both WT and 3xTg-AD astrocytic density, and differences between genotypes in both subiculum hippocampal and cortical regions. *n* = 5–6 mice per genotype/age/sex. Data are represented as mean ± SEM. *p ≤ 0.05, ^**^p ≤ 0.01, ^***^p ≤ 0.001.

To investigate changes in glial cells in the brains of 3xTg-AD mice, sections of wildtype and 3xTg-AD mice at all timepoints were immuno-stained using antibodies against the microglial marker IBA1 (Figure 7A), or astrocytic markers GFAP and S100β (Figure 7G). An increase in microglial density in the subiculum accompanied the presence of dense core plaques in 18-month-old female 3xTg-AD mice (Figures 7B–D). Plaques are not observed in the cortex of 3xTg-AD mice at any age (up to 18 mo of age), and no increase in microglial density was seen within cortical regions. An age-associated reduction in microglial density is observed in 12 and 18-month old WT females compared to male mice (Figures 7E,F). In this study, 18-month-old male 3xTg-AD mice also have reduced densities of microglia in the visual cortex compared to WT controls (Figure 7F).

Densities and distribution of homeostatic (S100β +ve) and reactive (GFAP +ve) astrocytes were also measured by immunostaining. S100β is a transcription factor localized in the nucleus of all astrocytes while GFAP is expressed in both hippocampal astrocytes and “reactive” astrocytes in the cortex (Figure 7G). Within the subiculum, homeostatic astrocytes (S100β +ve) populations are unchanged across all timepoints whereas reactive (GFAP +ve) astrocytes accumulate around Thio-S^+^ plaques (Figure 7H). The density of S100β^+^ astrocytes in the subiculum hippocampal region across each timepoint was stable (Figures 7I,J) whereas the density of GFAP +ve astrocytes increases at the 18-month timepoint (Figures 7M,N). Interestingly, S100β^+^ astrocyte densities decrease with age in cortical regions of both 3xTg-AD and wildtype mice with a general trend particularly in both 3xTg-AD and WT female mice at 18 months (Figures 7K,L). As expected, no reactive astrocytes are detected in cortical regions due to the lack of Thio-S^+^ plaques; therefore, no quantification was performed for GFAP immunostaining in cortical regions.

### Age-Dependent Change in Perineuronal Net Volume and Loss of Parvalbumin^+^ Interneurons in 3xTg-AD Brains

**FIGURE 8:**
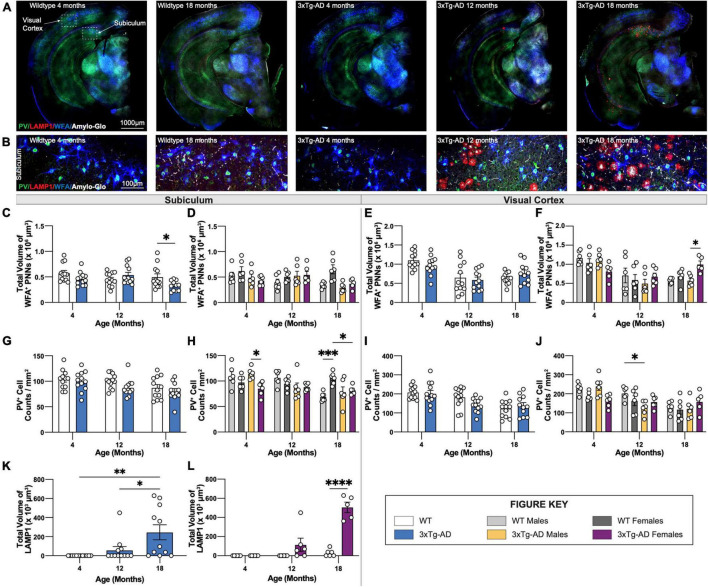
PNN and LAMP1. Perineuronal nets (PNNs) and parvalbumin (PV) interneurons were assessed with immunostaining using WFA and PV antibody, while lysosomes were assessed with immunostaining using LAMP1 antibody. **(A)** Representative images of WT (4-, 18-month) and 3xTg-AD mice (4-, 12-, 18-month) stained with PV/LAMP1/WFA/Amylo-Glo. **(B)** Representative images of WFA^+^ PNNs surrounding PV^+^ neurons around LAMP1^+^ lysosomes in subiculum hippocampal regions of WT and 3xTg-AD mice across respective timepoints. **(C–F)** Age-related change in total volume of WFA^+^ PNNs in 3xTg-AD mice. **(G–J)** Immunostaining for PV^+^ neurons demonstrate age-related changes in the density of PV^+^ neurons in both WT and 3xTg-AD mice, and differences between genotypes in subiculum and cortical regions. *n* = 5–6 mice per genotype/age/sex. **(K,L)** LAMP1 immunostaining reveals age- and sex-associated increases in the total volume of LAMP1^+^ lysosomes in the subiculum brain region of 3xTg-AD mice. *n* = 6 mice per genotype/age/sex. Data are represented as mean ± SEM. *p ≤ 0.05, ^**^p ≤ 0.01, ^***^p ≤ 0.001, ^****^p ≤ 0.0001.

We previously reported plaque-induced and microglia-mediated loss of perineuronal nets (PNNs) in the 5xFAD mouse model of AD as well as in human AD prefrontal cortex (Crapser et al., 2020). Age-associated changes in PNNs were investigated in hippocampal and cortical regions of 3xTg-AD and wildtype mice at each timepoint using the lectin *Wisteria floribunda agglutinin* (WFA), as well as parvalbumin (PV) which stains parvalbumin^+^ (PV^+^) interneurons, LAMP1 – a marker of dystrophic neurites, and Amylo-Glo for dense core plaques (Figures 8A,B). In accordance, 18-month-old 3xTg-AD mice show significant reductions in PNN volume in the subiculum (Figures 8C,D) coincident with the appearance of plaques, while no changes are seen in the visual cortex (Figures 8E,F) which lack plaques. Interestingly, in WT mice females maintain PNN volumes across their lifespans in the subiculum, while male mice show age-dependent reductions (Figures 8C,D).

In 5xFAD mice, loss of PNNs preceded loss of parvalbumin^+^ (PV^+^) interneurons (Crapser et al., 2020). Likewise, significant reductions in PV^+^ interneurons are seen in the subiculum of 18-month-old female 3xTg-AD compared to WT mice (Figures 8G,H). Notably, mirroring the age-related loss of WFA^+^ PNN’s in male WT mice is an age-related loss of PV^+^ neurons, which are maintained in female mice (Figures 8G,H). Age related reductions in PV^+^ interneurons in the visual cortex are seen in both male and female mice, but no additional reductions in 3xTg-AD mice (Figures 8I,J), presumably due to the lack of plaque pathology in that region.

### Age-Dependent Accumulation of Dystrophic Neurites in 3xTg-AD Hippocampus

Dystrophic neurites surround dense core plaques, which can be visualized through immunostaining with LAMP1. Immunofluorescence staining was performed using an antibody against LAMP1 and Amylo-Glo, a fluorescent tracer for Aβ plaques, on brain sections of 3xTg-AD and wildtype mice at 4-, 12-, and 18-month timepoints (Figures 8A,B). Neither LAMP1^+^ dystrophic neurites nor Amylo-Glo^+^ plaques accumulate in wildtype mice. In contrast, 3xTg-AD mice show the presence of dystrophic neurites alongside plaques within subiculum brain regions (Figure 8B). Quantifying the total volumes of LAMP1 staining reveals significant age-associated increase in dystrophic neurites in 3xTg-AD mice, which is mostly due to female 3xTg-AD mice (Figures 8K,L). As expected, this pattern mirrors the age-associated increase of Thio-S^+^ dense core plaques in female 3xTg-AD mice.

### Changes in Gene Expression Found in 3xTg-AD Brains

Bulk tissue gene expression was measured via RNA-seq, from micro-dissected hippocampi, and can be explored in an interactive and searchable fashion at https://modeladexplorer.org/. Comparisons between bulk tissue and single cell/single nucleus RNA-seq from 3xTg-AD brains are explored extensively in a companion publication (Balderrama-Gutierrez et al., 2021).

### Comparison of 3xTg-AD and 5xFAD Mouse Models

**FIGURE 9:**
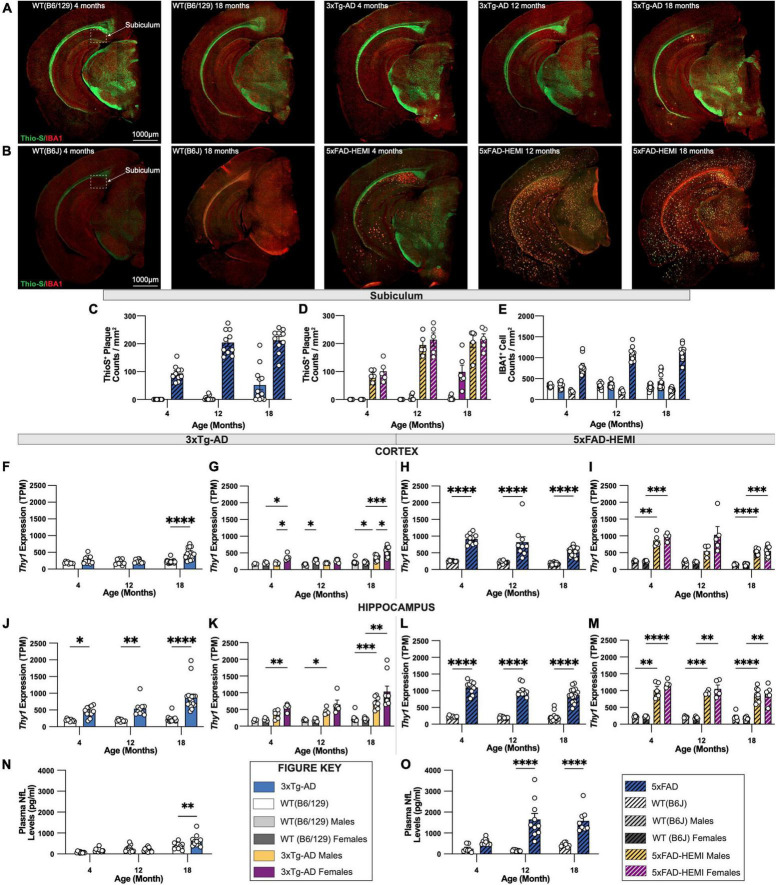
Comparison of fibrillar amyloid plaque accumulation in 3xTg-AD and 5xFAD mice. **(A)** Representative images of brain hemispheres of WT(B6;129) at 4- and 18-month and 3xTg-AD mice at 4-, 12-, 18-month stained with Thio-S/IBA1. **(B)** Representative stitched brain hemispheres of WT(B6J) at 4- and 18-month and hemizygous 5xFAD mice at 4-, 12-, 18-month stained with Thio-S/IBA1. **(C,D)** Quantification for density of Thio-S^+^ plaques in subiculum hippocampal regions in 3xTg-AD and 5xFAD mice showed differences in plaque burden between mouse models and sexes. **(E)** Quantification for IBA1 immunostaining for microglia in subiculum hippocampal regions reveals age-related differences between mouse models. **(F,G)** TPM values for *Thy1* expression in micro-dissected cortical lysates revealed an age-associated increase in 3xTg-AD, as well as sex-differences driven by female mice. **(H,I)** TPM values for *Thy1* expression in micro-dissected cortical lysates in 5xFAD mice displays a significant increase compared to B6J wildtype mice, yet no sex differences were observed. **(J,K)** TPM values for *Thy1* expression in micro-dissected hippocampal lysates demonstrate higher *Thy1* expression in 4-, 12-, and 18-month-old 3xTg-AD mice. **(L,M)** TPM values for *Thy1* expression in micro-dissected hippocampal lysates display a similar increase in *Thy1* expression compared to B6J wildtype controls with no sex differences within groups. **(N,O)** Quantification of neurofilament-light chain (NfL) levels of plasma from 3xTg-AD and 5xFAD using Mesoscale Singleplex technology demonstrated an age-related increase in 3xTg-AD mice at only the 18-month timepoint **(N)**, while age-related increases were found in 5xFAD mice at both 12-month and 18-month timepoints **(O)**. *n* = 5–10 mice per genotype/age/sex. Data are represented as mean ± SEM. *p ≤ 0.05, ^**^p ≤ 0.01, ^***^p ≤ 0.001, ^****^p ≤ 0.0001.

To enable a comparison of an onset of Alzheimer’s-related histopathology between two established animal models of AD, representative sections of 3xTg-AD and 5xFAD mice with their respective wildtype B6/129 and B6J controls were immuno-stained for Thio-S^+^ dense core plaques and IBA1^+^ microglia. Representative images of brains of each wildtype mouse display minimal differences between B6/129 and B6J controls. Abundant accumulation of Thio-S^+^ dense core plaques is apparent in hemizygous 5xFAD mice at 4-months of age compared to 3xTg-AD mice at 18-months of age (Figures 9A,B). Analysis of subiculum hippocampal regions reveals an increase in the density of Thio-S^+^ plaques in hemizygous 5xFAD mice compared to 3xTg-AD (Figures 9C,D). These differences are also observed in the microgliosis of IBA1^+^ cells surrounding dense core Thio-S^+^ plaques. As expected, a significant increase in IBA1^+^ microglia density accompanies the presence of Thio-S^+^ plaques in hemizygous 5xFAD mice as early as 4-months of age versus 18-month-old 3xTg-AD mice (Figure 9E).

We explored *Thy1* promotor expression, which drives the transgenes in both 3xTg-AD and 5xFAD mice [data derived from Balderrama-Gutierrez et al. (2021)] and (Forner et al., 2021, respectively. *Thy1* expression was elevated in 3xTg-AD cortex relative to wild-type *Thy1* (which does not drive a transgene but sets the background level) only at 18 months of age, consistent with the lack of pathology that we observe there (Figure 9F). Sex differences were also observed with female mice showing stronger *Thy1* expression than males in 3xTg-AD mice at 4 and 18 months of age (Figure 9G). On the other hand, *Thy1* expression in the cortex of 5xFAD mice was substantially elevated relative to wild-type mice from 4 months of age, and at far higher levels than in the 3xTg-AD mice at 18 months of age (Figures 9H,I), again consistent with the high plaque load seen in 5xFAD mice. 3xTg-AD show increased *Thy1* expression in the hippocampus relative to wild-type mice at all ages, and at higher levels than in the cortex (Figure 9J), consistent with the presence of pathologies in the hippocampus at advanced ages, but not the cortex. While *Thy1* levels trended higher in females than males, this did not reach significance (Figure 9K). *Thy1* levels in 5xFAD hippocampus were again far higher than those found in 3xTg-AD mice (Figures 9L,M), again explaining the dramatic differences in pathology seen in these two lines.

Recently, plasma levels of neurofilament-light chain (NfL) from AD patients have been demonstrated to correlate strongly with amyloid plaque load and risk of developing cognitive dementia (de Wolf et al., 2020). Therefore, as a potential biomarker to assess and monitor progression of AD, we quantified NfL levels in plasma from either 3xTg-AD or 5xFAD mice, and their wildtype B6/129 or B6J controls, respectively. Notably, 5xFAD mice showed a robust increase in plasma NfL at all ages, increasing and correlating with plaque load (Figure 9O). Similarly, plasma NfL from 3xTg-AD mice showed increases relative to their controls only at 18 months of age, when some plaques are present in the subiculum (Figure 9N). Furthermore, increased plasma NfL at 18 months was far less than that seen in 5xFAD mice, even at their 4-month age, suggesting that plaque load appears to be the major driver of plasma NfL in both of these models (Figures 9N,O).

## Discussion

We have evaluated the AD-related pathogenesis in current 3xTg-AD mice and highlight the drift that has occurred over the past two decades in terms of development of pathology. Since our original report in 2003 (Oddo et al., 2003), the development of pathology has been substantially delayed, and a sex difference has manifested, and thus this study represents an updated characterization of the current 3xTg-AD mice. As originally described, we show that 3xTg-AD mice develop both plaques and tangles in an age-related fashion, but that the timing of plaque development has been substantially retarded since the line was first produced. Also striking has been the emergence of profound sex differences in the development of both plaques and tangles, with these pathologies only emerging in female mice. Previously, we reported no sex differences in terms of pathology, but the development of deficits in female mice on stressful behavioral tasks (Clinton et al., 2007). In line with our updated findings, increased Aβ levels have been reported in female 3xTg-AD mice at 12-months of age (Carroll and Pike, 2008; Carroll et al., 2010; Yang et al., 2018; Creighton et al., 2019), and 18-months of age (Perez et al., 2011; Bories et al., 2012; Vandal et al., 2015). These changes in phenotype are the result of drift that has occurred in ours, and others, colonies. It should be noted that drift may have occurred differently in different colonies of 3xTg-AD mice, and thus other timings of pathology may exist (i.e., Belfiore et al., 2019); however, this present study uses mice from the current colony at The Jackson Labs (stock number: 004807). Increased pathology in female mice is also seen in the 5xFAD mouse model, which uses the same *Thy1* mini-gene regulating expression of the cDNA’s in the transgenes. The promoter in this *Thy1* mini-gene was found to contain an estrogen response like element that can produce greater expression in females (Sadleir et al., 2015), which we recently replicated with our phenotyping efforts (Forner et al., 2021). Hence, the female sex-bias in pathology in both 3xTg-AD and 5xFAD AD models is likely due to increased expression from the *Thy-1* mini-gene, rather than reflecting some inherent female-specific bias in AD susceptibility as found in the human population. Female 3xTg-AD mice develop both plaques and tangles, and their appearance coincides with loss of the extracellular matrix structures that surround mainly inhibitory neurons known as perineuronal nets, loss of parvalbumin^+^ interneurons, and increased NfL levels in plasma. Our findings regarding the loss of perineuronal nets followed by loss of parvalbumin^+^ interneurons in the hippocampus of 3xTg-AD mice corroborate prior findings implicating Aβ-dependent microglia activation facilitating the same pathology originally reported in the hippocampus of 5xFAD mice and AD patients (Crapser et al., 2020). Additionally, our observation of plaque-dependent increases in plasma NfL in both 3xTg-AD and 5xFAD mouse models adds further evidence for the potential use of NfL as a biomarker to monitor progression of Aβ pathology in future animal models and AD patients (de Wolf et al., 2020).

While the search for an “ideal” animal model of late-onset Alzheimer’s disease is ongoing, it is clear that the model of choice for one’s scientific investigation will largely depend on which models best address their specific hypothesis-driven research questions. To navigate the innumerable different AD animal models available, a standardized deep-phenotyping characterizations of current and future animal models will be a useful tool to compare advantages and limitations unique to each model. Here, we demonstrate the utility of one such deep-phenotyping pipeline using the 3xTg-AD mouse model, in tandem with a prior characterization of the 5xFAD mouse model (Forner et al., 2021). The fact that these pathologies in the brains of 3xTg-AD mice develop slowly, and in an age-related fashion may be advantageous to studies that focus on late-onset AD and on the interactions between amyloid and tau pathologies. This finding contrasts with the aggressive amyloidosis models such as the 5xFAD mice, which produce magnitudes greater plaques from young ages (4+ months) and represent an excellent model for studying the effects of plaques on the brain (Oakley et al., 2006; Forner et al., 2021). Notably, these mouse models are only two among the many AD animal models currently available to the Alzheimer’s research community in addition to new strains that implement LOAD genetic risk variants generated by MODEL-AD. Therefore, approaches like the robust, in-depth phenotyping characterization pipelines reported here will better equip researchers with standardized comparisons regarding the benefits and limitations of characterized animal models with the goal of identifying the most optimal animal model specific to their own investigation and research questions.

## Data Availability Statement

The datasets presented in this study can be found in online repositories. The names of the repository/repositories and accession number(s) can be found below: Sage Synapse (https://www.synapse.org/#!Synapse:syn25878481); gene expression data for 3xTg-AD mice - doi: 10.7303/syn26524615 (https://www.synapse.org/#!Synapse:syn26524615); gene expression data for 5xFAD mice - doi: 10.7303/syn23628482 (https://www.synapse.org/#!Synapse:syn23628482).

## Ethics Statement

The animal study was reviewed and approved by UC Irvine Institute Animal Care and Use Committee.

## Author Contributions

DJ, KT, JP, EH, EK, CC, SF, SK, CB, MH, DM, NR, and JA performed the experiments. DJ, JP, EH, EK, SF, SK, GM, AG-A, and KG analyzed the data. DJ and KG wrote the manuscript with contributions from all authors. JN, MW, GRM, AM, AT, FL, and KG provided advice on the study design, managed the project, and contributed to the manuscript. All authors contributed to the article and approved the submitted version.

## Conflict of Interest

The authors declare that the research was conducted in the absence of any commercial or financial relationships that could be construed as a potential conflict of interest.

## Publisher’s Note

All claims expressed in this article are solely those of the authors and do not necessarily represent those of their affiliated organizations, or those of the publisher, the editors and the reviewers. Any product that may be evaluated in this article, or claim that may be made by its manufacturer, is not guaranteed or endorsed by the publisher.

## Footnotes

1
https://www.model-ad.org/

2
https://modeladexplorer.org/

3
https://adknowledgeportal.synapse.org

4
https://adknowledgeportal.org/DataAccess/Instructions

5
https://www.synapse.org/#!Synapse:syn25878481

6
https://www.synapse.org/#!Synapse:syn26524615

7
https://www.synapse.org/#!Synapse:syn23628482
