# Supplementary material for: Systematic Phenotyping and Characterization of the 3xTg-AD Mouse Model of Alzheimer’s Disease
Source: Front Neurosci. 2022 Jan 24;15:785276. doi: 10.3389/fnins.2021.785276 (PMC8818877; doi:10.3389/fnins.2021.785276)
Supplement: Supplementary file 1 [file Data_Sheet_1.pdf]

The “ARRIVE Essential 10” Responses

Manuscript: Systematic phenotyping and characterization of the 3xTg-AD mouse model of Alzheimer’s Disease

1. Study Design

For each experiment, provide brief details of study design including:

- ❑ The groups being compared, including control groups. If no control group has been used, the rationale should be stated
    - *Experimental homozygous 3xTg-AD were compared with non-transgenic C57B6/129 littermates (I.e. wild-type controls). Throughout the text, 3xTg-AD homozygous animals will be referred to as “3xTg-AD” and closely related non-transgenic and Psen +/+ mice are referred to as “wildtype” (WT) controls. Additionally, 5xFAD hemizygous mice were used with non-transgenic C57BL/6J littermates (I.e. wildtype controls)*
- ❑ The experimental unit (e.g. a single animal, litter, or cage of animals)
    - *The experimental unit in this manuscript are single animals.*

2. Sample Size

- ❑ Specify the exact number of experimental units allocated to each group, and the total number in each experiment. Also indicate the total number of animals used.
    - *For behavioral, histological, and biochemical analysis, six male and six female mice were used for each 3xTg-AD and WT(B6129) group with ages at 4-, 12-, and 18-month timepoints. For hippocampal slice preparation and LTP recording, five male and five female mice were used for each 3xTg-AD and WT (B6129) group at 4-, 12-, and 18-months of age. For 5xFAD histological analysis, five male and five female mice were used for each 5xFAD and WT (B6J) group with ages as 4-, 12-, and 18-month timepoints.*
- ❑ Explain how the sample size was decided. Provide details of any a priori sample size calculation, if done.
    - *We have determined that 6 animals per sex per timepoint and per genotype is required to fulfill all of the endpoints required for the in-depth phenotyping planned. This includes:*
    - *Behavior and cognitive testing - power analyses and prior experience has informed us that n=6 per group is required for statistical significance in these tasks. Given the focus on sex differences in AD, and research in general, we therefore require 6 males and 6 females per timepoint per genotype. Tissue from these mice (and the additional 6 mice per sex) will then be used for 1) LTP measurements (n= 5 hemispheres / sex), 2) biochemistry (n=6 hemispheres / sex), 3) immunohistochemistry (n=6 hemispheres / sex), and 4) gene expression via RNA-seq (n=6 hemispheres / sex)*

3. Inclusion and Exclusion criteria

- ❑ Describe any criteria used for including and excluding animals (or experimental units) during the experiment, and data points during the analysis. Specify if these criteria were established a priori. If no criteria were set, state this explicitly.
  - *No criteria was established a priori to exclude animals during the experiment, and data points during the analysis.*
- ❑ For each experimental group, report any animals, experimental units or data points not included in the analysis and explain why. If there were no exclusions, state so.
  - *No animals or data points were excluded from data analysis.*
- ❑ For each analysis, report the exact value of n in each experimental group.
  - *For 3xTg-AD LTP recording, there was an n = 5 per age/sex/genotype.*
  - *For 3xTg-AD histological analysis, there was an n = 6 per age/sex/genotype. For 5xFAD histological analysis, there was an n = 5 age/sex/genotype.*
  - *For 3xTg-AD biochemical analysis, there was an n = 6 per age/sex/genotype.*

4. Randomization

- ❑ State whether randomisation was used to allocate experimental units to control and treatment groups. If done, provide the method used to generate the randomisation sequence.
  - *Randomisation was not used to allocate experimental units to control and treatment groups. Allocation of experimental units was dependent on genotype of the animal.*
- ❑ Describe the strategy used to minimise potential confounders such as the order of treatments and measurements, or animal/cage location. If confounders were not controlled, state this explicitly.
  - *To minimize environmental confounders, animals were grouped housed in autoclaved individual ventilated cages containing autoclaved corncob bedding and two autoclaved 2" square cotton nestlets plus a LifeSpan multi-level environmental enrichment platform. Tap water and food were provided ad libitum.*

5. Blinding

- ❑ Describe who was aware of the group allocation at the different stages of the experiment (during the allocation, the conduct of the experiment, the outcome assessment, and the data analysis).
  - *Shimako Kawauchi and Stefania Forner were aware of the group allocation when breeding and allocating mice within each experimental group. During the conduct and outcome assessment of each experiment, Stefania Forner was aware of the group allocation of each mouse. During the data analysis, Stefania Forner, Jimmy Phan, Edna Hingco, and Eniko Kramar were aware of the group allocation of each mouse.*

6. Outcome Measures

- ❑ Clearly define all outcome measures assessed (e.g. cell death, molecular markers, or behavioural changes).

  - *For electrophysiology analysis, outcome assessments include measuring changes in field excitatory postsynaptic potential (fEPSPs) and recordings of long-term potentiation (LTP).*

  - *For histological analysis, outcome assessments include Thioflavin-S and Amylo-Glo stains for accumulation of dense-core plaques and Gallyas' Silver stains for accumulation of neurofibrillary tau tangles. Additionally, immunostaining was used to detect changes in the presence of ionized calcium-binding adapter molecule 1 (IBA1), $Aβ_{1-16}$ (6e10), glial fibrillary acidic protein (GFAP), S100β, Fox 3 protein (NeuN), Ctip2, lysosome-associated membrane protein 1 (LAMP1), human tau (HT7), poly-tau, phospho-tau Ser202 (AT8), phospho-tau Thr217 (pTau217), Wisteria floribunda agglutinin (WFA), and parvalbumin (PV).*

  - *For biochemical analysis, outcome assessments include detecting changes in levels of soluble and insoluble human Aβ using V-PLEX Aβ Peptide Panel 1 plates.Quantitative biochemical analysis of total tau and phosphorylated tau-231 (pTau231) in soluble fractions were also obtained using the Phospho(Thr231)/Total Tau Kit (K15121D-1; Meso Scale Discovery, Rockville, MD). Finally, quantitative biochemical analysis of neurofilament-light chain (NfL) in plasma was performed using the R-Plex Human Neurofilament L Assay (K1517XR-2; Meso Scale Discovery, Rockville, MD).*

- ❑ For hypothesis-testing studies, specify the primary outcome measure, i.e. the outcome measure that was used to determine the sample size.

  - *No primary outcome measure was used to determine the sample size.*

7. Statistical Measures

- ❑ Provide details of the statistical methods used for each analysis, including software used.

  - *Statistical analyses were performed using the GraphPad Prism v.9 software. Behavioral, immunohistochemical, and biochemical data were analyzed using Student's t-test, one-way ANOVA, or two-way ANOVA. Bonferonni-Šídák and Tukey's post hoc tests were utilized to examine biologically relevant interactions from the two-way ANOVA. *$p \leq 0.05$, ** $p \leq 0.01$, ***$p \leq 0.001$, ****$p \leq 0.0001$. Statistical trends are accepted at $p < 0.10$ (#). Data are presented as raw means and standard error of the mean (SEM).*

- ❑ Describe any methods used to assess whether the data met the assumptions of the statistical approach, and what was done if the assumptions were not met.

  - *No method was used to assess whether data met the assumption of the statistical approach.*

8. Experimental Animals

- ❑ Provide species-appropriate details of the animals used, including species, strain and substrain, sex, age or developmental stage, and, if relevant, weight.

- *Homozygous 3xTg-AD (B6;129-Tg(APPSwe,tauP301L)1Lfa Psen1^{tm1Mpm}/Mmjax mice were obtained from a closed colony maintained in the laboratory of F.M.L.. In 2018, this stock was genotyped using SNP markers and shown to be homozygous for 129X1/129S1 alleles at ~35% of the genome, homozygous for C57BL/6 alleles at ~ 50% of the genome and heterozygous for alleles of 129X1/129S1 and C57BL/6 at ~ 15% of the genome. Experimental outcome measures were observed in male and female 3xTg-AD and WT mice at 4-, 12-, and 18-months of age.*

❑ Provide further relevant information on the provenance of animals, health/immune status, genetic modification status, genotype, and any previous procedures.

- *At that time, qPCR analysis of the APPSwe and TAUP301L cDNA's in 3xTg-AD mice from the LaFerla colony and those from Jackson Laboratory (Stock # 34830) showed a similar relative copy number for each cDNA. Experimental and control mice for this study were generated as follows. First, sperm from 3xTg-AD homozygous animals from the LaFerla colony was used to fertilize oocytes from B6129SF2/J mice (Jackson Laboratory, Stock # 101045) and zygotes were transferred to pseudopregnant dams. F1 offspring were genotyped to verify heterozygosity for the co-integrated Thy1-APPSwe and Thy1-TAUP301L transgene array at ~ 87.9Mb on chromosome 2, and the I145V/M146V mutations in Psen1 on chromosome 12. F1 heterozygous mice were intercrossed to generate F2 offspring that were genotyped to identify animals homozygous for both the transgene array and Psen1 mutations, (i.e. experimental 3xTg-AD homozygotes) or Psen1$^{+/+}$ and non-transgenic (i.e. wild-type control).*

9. Experimental Procedures

For each experimental group, including controls, describe the procedures in enough detail to allow others to replicate them, including:

❑ What was done, how it was done and what was used.

- *Addressed in "Materials and Methods" Section.*

❑ When and how often.

- *Addressed in "Materials and Methods" Section.*

❑ Where (including detail of any acclimatisation periods).

- *Addressed in "Materials and Methods" Section.*

❑ Why (provide rationale for procedures).

- *Addressed in "Materials and Methods" Section.*

10.Experimental Procedures

For each experiment conducted, including independent replications, report:

❑ Summary/descriptive statistics for each experimental group, with a measure of variability where applicable (e.g. mean and SD, or median and range).

    - *Summary statistics is presented as mean ± standard error mean (SEM) for each experimental group*

- ❑ If applicable, the effect size with a confidence interval.
    - *Addressed in "Materials and Methods" Section*.
